# Microplastics in Commercial Fishes and By-Catch from Selected FAO Major Fishing Areas of the Southern Baltic Sea

**DOI:** 10.3390/ani13030458

**Published:** 2023-01-28

**Authors:** Paulina Piskuła, Aleksander Maria Astel

**Affiliations:** Environmental Chemistry Research Unit, Institute of Biology and Earth Sciences, Pomeranian University in Słupsk, 76-200 Słupsk, Poland

**Keywords:** microplastics, contamination, ingestion, fish, Baltic Sea, FT-IR analysis

## Abstract

**Simple Summary:**

A variety of plastics are produced and used in packaging, construction, transport, medicine, electrical, and clothing industries. Despite many advantages, plastics pose a serious threat to ecosystems, mainly due to the high generation of waste. Microplastics of diameter below 5 mm can either result from the degradation and weathering of larger items (secondary microplastics), or from the direct discharge of materials originally manufactured at that size (primary microplastics). Many of them are released directly or indirectly to fresh and salty water reservoirs. The size of microplastic particles and appealing coloration allow for easy ingestion by fish, which in turn leads to physical damage to the gastrointestinal tract or tissue and organ toxicity. This study aimed to assess the presence of microplastics in organs of fish from the southern Baltic Sea acquired as a raw material of commercial value (for food processing plants) and as by-catch. Microplastics abundance in gills, digestive tract and liver ranged from 1 to 12 items per fish, with an average of around 4. Blue fibers were prevalent among other forms such as particles and pellets. Fish guts and gills can be regarded as important organs in monitoring ecological risks for fishes exposed to contamination with microplastics.

**Abstract:**

According to recent world wide studies, microplastics (MPs) have been found in many fish species; however, the majority of research has focused only on the gastrointestinal tract, neglecting edible organs. This study aimed to assess the presence of microplastics in the non-edible (gills, digestive tract) and edible organs (liver) of three commercial fish species and twoby-catch species from the southern Baltic Sea. Fish (*Clupea harengus, Gadus morhua, Platichthy sflesus, Taurulus baublis, Cyclopterus lumpus*) were caught in 108 and 103 FAO Fishing Zones belonging to the Polish fishing zone. The abundanceof MPs ranged from 1 to 12 items per fish, with an average of 4.09 items. MPs were observed in different organs, such as the liver, gills, and digestive tract of all five tested species. MPs recognized as fibers were the most abundant. Other shapes of polymers found in fish organs were pellets and particles of larger plastic pieces. The dominant color of the MPs was blue, but there were also red, black, transparent, yellow, green, and white items found. According to dimensions, dominant MPs were between 0.1 and 0.5 mm in size. The chemical characterization of polymers accomplished by the use of Fourier Transform Infrared (FT-IR) Spectroscopy demonstrated the abundance of cellophane, polyamide, polyethylene, polypropylene, polyethylene terephthalate, polyvinyl propionate, polyacrylonitrile, and polyester.

## 1. Introduction

The contamination of aquatic organisms with polymer-based plastic items is an increasing problem for global food safety [1]. Commercial fish and seafood species ingest MPs due to a range of factors and behaviors [2,3]. Plastic items have also been identified in edible seaweed [4]. Ingestion of plastic items can lead to physical damage to the gastrointestinal tract as well as to malfunction of tissue and organs due to the toxic effect of chemicals released from plastic. Among a huge variety of xenobiotics, MPs can release toxic plastic additives, such as phthalates, bisphenol-A, polybrominated diphenyl ethers, nonylphenol, and dyes into the body of any organism that ingested it with food or by any other mechanism [5]. A high sorption ability coupled with strong hydrophobicity enable MPs to easily adsorb organic chemicals in the environment [6]. Studies have shown that MPs present in sea-water reservoirs accumulate about a two to six orders of magnitude higher load of organic pollutants than sediment or water [7]. Organics that are widespread in many aquatic ecosystems usually have a long retention time and cause toxic effects of various intensities [8]. Many authors [9] have proven the presence of persistent organic pollutants (POPs) in polypropylene (PP), polyethylene (PE), and polystyrene (PS) items along the Portuguese coast, including polychlorinated biphenyls (PCBs), polycyclic aromatic hydrocarbons (PAH), and dichlorodiphenyltrichloroethane (DDT). Moreover, laboratory experiments with seawater and freshwater used as solvents have shown that PE, PS, PP, polyvinyl chloride (PVC), and polyamide (PA) can adsorb antibiotics [10]. The spectrum of negative effects caused by organic pollutants towards organisms inhabiting water reservoirs includes the inhibition of the microalgae growth [11], physical injuries of crustacea [12], musculoskeletal changes of *Danio rerio* larvae [13], feminization of fish [14], etc. Diclofenac at a level of 5–50 µg/L affects the parameters of kidneys, gills, and fish resistance [14]. Ibuprofen can accumulate in fish tissues and change cellular responses in the liver, kidneys, and gills, as well as disrupt the endocrine system by altering aromatase activity, which then affects the balance of sex hormones [15].

Environmental monitoring campaigns as well as laboratory tests have shown the accumulation of heavy metals in MPs. Metals such as Al, Fe, Mn, Cu, Ag, Zn, Co, Mo, Sb, Sn, and Pb were found on polymer items in a variety of both fresh and salty water ecosystems. Many experiments have shown higher concentrations of metals on the surface of MPs than in the surrounding sediments and seawater [16,17,18]. Some controlled tests have shown the adverse effects of MPs in combination with heavy metals on aquatic fauna [19,20].

MPs are ubiquitous in the aquatic environment. They occur in inland lakes [21], rivers [22,23], deep-sea environments [24,25], on islands [26], in sub-arctic areas [27], and the seas and oceans [28,29].

Fish and fish products are very important ingredients in human nutrition. They are a source of valuable nutrients, especially omega-3 fatty acids. According to Food and Agriculture Organization (FAO) as well as World Health Organization (WHO) experts, regular consumption of fish (1–2 times a week) is recommended for the prevention of ischemic heart disease [30]. Unfortunately, over 80% of Poles eat a maximum of one fish meal a week or less [31]. Eating fish meat or edible organs, such as the liver, is beneficial, but when recommending increasing its share in the diet, you should also take into account the potential risks resulting from the presence of substances that have a detrimental effect on the human body.

Plastic items can be intentionally, accidentally, or randomly ingested by fish [32]. The quantification of the ingestion of MPs by aquatic organisms is very well documented [1,33,34], however, occasional studies have focused on the analysis of MPs in fish caught in specific fisheries. Many research results have suggested that MPs accumulated in the digestive tract are excreted [35,36], however, there is a need to understand the migration of plastic items into tissues and organs.

Research on the occurrence of micro- and nano-plastics in the ichthyofauna of theNorth Sea is well documented [37,38,39,40], however, to our knowledge, there are only a few papers concerning the abundance of MPs in fish caught in the northern Baltic Sea [37,41,42,43] and only one publication concerning fish caught in the southern waters of the Baltic Sea (BS) [44]. The latter study included the gills and digestive tract of cod (*Gadus morhua*) and herring (*Clupea harengus*); however, the number of tested samples was quite small. Therefore, there is an urgent need to monitor the abundance as well as to determine the morphometric features and chemical types of polymer items present in the fish caught in the Polish fishing zones of the South Baltic Sea.

The purposes of this study were to: (i) assess the presence of MPs in the digestive tract, liver, and gills of fish caught in the southern BS, (ii) assess the type and the morphometric features of the MPs, and (iii) characterize the identified MPs by the use of FT-IR spectrometry.

## 2. Materials and Methods

### 2.1. Study Area

The Baltic Sea lies between 10°–30° E and 54°–66° N and constitutes one of the world’s largest brackish water areas. The surface salinity in the Baltic Sea does not exceed 10 psu, while the average salinity of the entire reservoir is around 7.5 psu [45]. Its basin is about 0.1% and 0.002% of the world’s ocean area and volume, respectively. The Baltic Sea belongs to shallow reservoirs, exhibiting a maximum depth of 460 m and a mean depth of 60 m [46]. The total Baltic Sea catchment area comprises 1,729,500 km^2^ being more than four times larger than the surface of the Baltic Sea. Its area, including the Kattegat, is approx 415,266 km^2^. It is surrounded by Poland, Germany, Denmark, Sweden, Norway, Finland, Russia, Estonia, Latvia, and Lithuania [47]. The Baltic Sea belongs to the group of the most polluted inland seas in the world, mainly due to limited water exchange with other seas and the huge impact of environmental stressors, such as eutrophication, overfishing, chemical and oil spills, dumped conventional warfare [48], marine litter, etc. Its ecosystem is influenced by activities in the coastal zone [49], in the sea itself [50], and by the indirect impact of the catchment area. The pollutants released to the Baltic Sea come from a variety of sources. Concerning the release of MPs, assessments made in Denmark, Norway, Germany, and Sweden have indicated that the emission from secondary sources dominates over primary ones. From the secondary sources, emission from tires, ship paints, footwear, road markings, textiles, personal care products, raw material loss, laundry, and boat hulls dominates; however, common to almost all sources is a big range of estimated values together with high uncertainty [51].

### 2.2. Commercial Value of Fish from the Baltic Sea

Avariety of fish species live in the Baltic Sea. In comparison with 120 species found in the adjacent North Sea in the Baltic Sea, 26 species of typical marine fish have been recorded.

In comparison with the North Sea, the number of commercial species is fewer; however, they are quite important for the worldwide range economy. Among others, these include pelagic herring (*Clupea harengus*) and sprat (*Sprattus sprattus*); demersal: cod (*Gadus morrhua*) and flatfish (*Platichthys flesus*); migratory: salmon (*Salmo salar*), sea trout (*Salmo trutta*) and eel (*Anguilla anguilla*). Non-commercial fish live mainly in the coastal zone, although they inhabit places of different depths. These include bullheads, several species of goby, stickleback, and others: lump (*Cyclopterus lumpus*), common seasnail (*Liparis liparis*), snakeskin (*Lumpenus lampretaeformis*), straightnose pipefish (*Nerophis ophidion*), and pine needle (*Syngnathus typhle*). A specific phenomenon in the Baltic Sea is the presence of freshwater fish. They appear in the estuaries of rivers, and brackish bays, often of vital importance to local fisheries. There are also typical species of two-environmental fish living in the Baltic Sea. Anadromous fish (salmon and sea trout) spend their adult lives in the sea, where they encounter favorable nutritional conditions. They spawn in the headwaters of Baltic streams and rivers. The eel is a catadromous fish, i.e., it migrates to the sea to spawn and spends its entire adult life in freshwater reservoirs [52].

### 2.3. Fish Characteristics and Specimen Collection Details

One pelagic (Baltic herring), one bentho-pelagic (Baltic cod), and three benthic fish species (flounder, long-spined bullhead, lumpfish) were included in this research.

Baltic herring (*Clupea harengus*) is one of the most dominant fish species in fish processing all over the world [53]. Its spawning takes place in bays characterized by warmer water and an abundance of food. In early life, it feeds on zooplankton in coastal regions [54], while as it grows nectobenthic organisms prevail in its food [55]. As an adult fish, it inhabits the deep water zone up to 250 m [56].

Baltic cod (*Gadus morhua*) is desired for economic purposes. It inhabits the entire reservoir of the BS, but usually, it feeds in the depths, where it creates shoals, especially in spring. In the remaining periods, it feeds alone or in smaller herds at various depths. Baltic cod is a predator fish that, apart from benthic invertebrates, mainly hunts on sprat and herring, which together account for about 85% of pelagic fish species in terms of biomass [57].

Flounder (*Platichthys flesus*) belongs to a group of fish with a laterally flattened body. It is a typical benthic fish, commonly found at depths up to 100 m [58]. Although it is a marine species, it is also found in rivers’ mouths and in brackish waters. Juveniles feed on plankton and insect larvae, while adults feed on mollusks, crustaceans, and small fish. It is valued in fishing and angling for its tasty meat [59].

Long-spined bullhead (*Taurulus Bubalis*) belongs to the Cottidae family and feeds mainly in coastal regions with rocky bottoms. It mainly inhabits the tidal zones of the BS north of the Gulf of Finland [60]. It is not a fish of high economic value. Adults lead a stationary lifestyle and rarely move along a given stretch of the rocky shore. The long-spined bullhead feeds on miceids, gammarus, tenths, polychaetes, mollusks, Ophiuchus, and fish [61].

Lumpfish (*Cyclopterus lumpus*) or lumpsucker is an economically important species. The meat of the females is unpalatable. Dyed and undyed lumpfish roe is sold as imitation caviar. It belongs to the group of benthic fish. It hunts crustaceans, small fish, and jellyfish [62].

**Figure 1:**
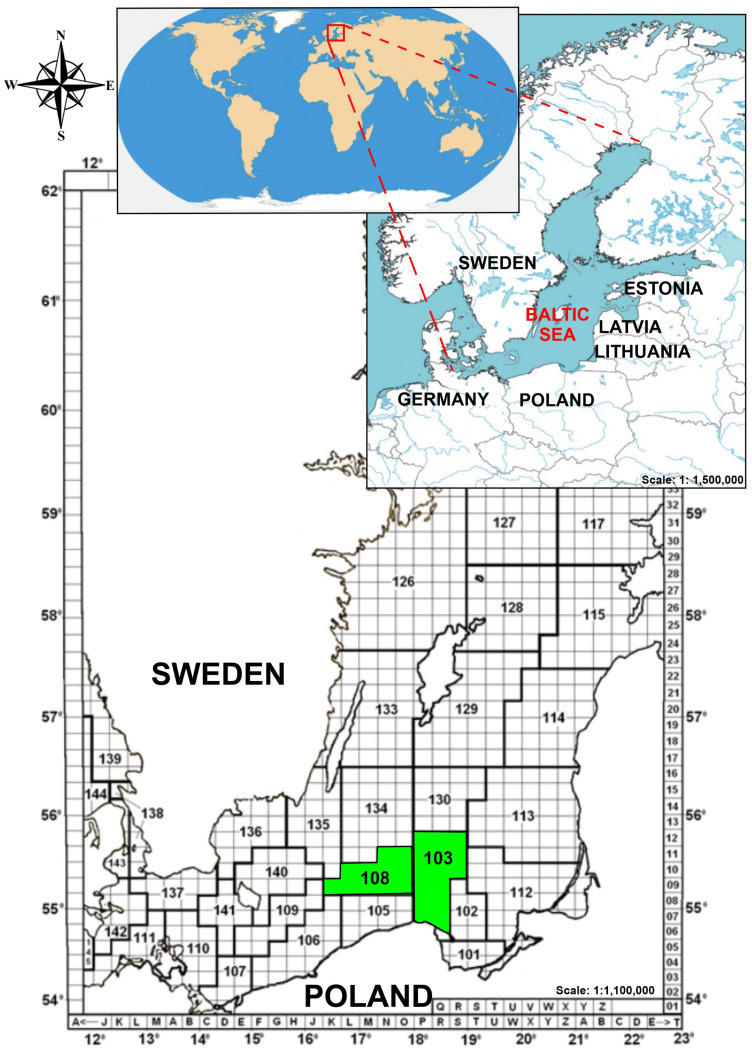
The sampling sites of fish collection in the south Baltic Sea.

Fish were collected by a commercial fishing vessel within area 27 FAO Major Fishing Areas for Statistical Purposes, sub-area IIID. Out of five species sampled two were by-catch. None of the sea-water samples were collected simultaneously by the crew of the vessel and this is why only some presumed explanation of the environmental conditions that play a possible role in the differentiation of the abundance of MPs in fish in this study and other research was justified. Figure 1 shows the catch sites.

Fish were identified up to the species level. The specimens were weighed using an analytical balance with Dibal Cely PS50-M (Spain) with an accuracy of ±0.01 g. The body length was measured from the tip of the snout (mouth closed) to the extended tip of the caudal fin. Finally, 87 collected fish samples were stored at −30°C for further analysis. The detailed characterization of the fish included in this study concerning their habitat as well as some morphometric features’ statistics is depicted in Table 1.

### 2.4. Microplastic Extraction and Identification

Before microscopic analysis, fishes were defrosted at room temperature and washed in deionized water (HLP 10 UV, Hydrolab, Poland). Tissues and organs (dorsal muscle, digestive tract, liver, and gill) were separated and weighted with a ±0.0001 g accurate digital analytical balance (Ohaus PX225D, Parsippany, NJ, USA). Dissected tissues and organs were placed in dry glass beakers. Since a digestion method proposed by Prata et al. [63], Karami et al. [64], or Dehaut et al. [65] did not allow effective sample digestion or caused a risk of the degradation of synthetic cotton fibers [66], acombined approach was followed for the process. A total of 10 mL of 10% KOH per gram of sample was added to all tissue samples, covered immediately with aluminum foil, and placed in an oven at 50 °C for 24 to 48 h to complete the digestion process. Once the complete dissolution of the viscera has finished, samples were placed in an ultrasonic bath (Inter Sonic IS-1, Olsztyn, Poland) with an ultrasound frequency of 40 kHz and a temperature of 55 °C for 15 min. Whatman No. 1001-090 filters of 11 μm pore size were used for assisted filtering. After the filtration was accomplished, cellulose filters were placed in Petri dishes and kept at an ambient temperature for 48 h to dry.

**Figure 2:**
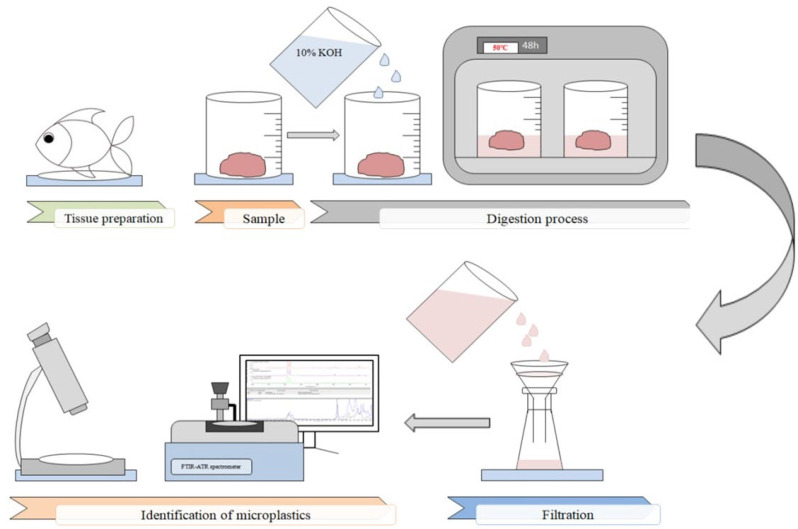
Schematic diagram of microplastic extraction and the identification protocol.

Afterward, the samples were examined in two stages: (i) by a stereoscope binocular (to determine the morphometric features of MPs items), (ii) and by an FT-IR spectrometer with an ATR accessory (to identify the chemical composition of the MPs). Sample filters were visually inspected under a stereomicroscope (Motic Zoom SMZ-161-BLED, Motic, Barcelona, Spain) with 0.75x−4.5x magnification equipped with 3W LED illumination and the Greenough optical system. Using a binocular connected to a tablet Moticam BTW8 (China) running under the control of the Android 5.0 operating system morphometric features of the samples were measured and archived using MotiConnect 1.5.9.10-build-171215 software which is a dedicated image processing Android app for Motic cameras. It includes image preview, capture, recording, editing, and measuring functions. Suspected plastic items were assessed in accordance with the protocols recommended by Hidalgo-Ruz et al. [67], Crawford and Quinn [68], and Zobkov and Esiukova [69]. Items with no visible tissue or cell structure of relatively uniform color distribution along the particle and fibers with homogenous diameters along their length were counted as MPs. The other objects were counted as minerals. An analogical procedure for the microscopic determination of MPs was applied by Wang et al. [70]. The identified MPs were divided into three morphological types: particles, pellets, and fibers, respectively. Based on the size, MPs were also divided into five groups of 0.005–0.1, 0.1–0.5, 0.5–1, 1–5, and >5mm. The color of the MPs was also recorded. Figure 2 shows a schematic diagram of the analytical protocol used in this study.

Once the microscopic assessment was finished, polymer items of the largest linear dimension higher than 500 μm were identified using an FT-IR spectrometer (Thermo Scientific, Nicolet iS5 with ATR diamond crystal, Waltham, MA, USA). Before spectroscopic analysis, the isolated plastic items were gently rinsed 5 times in deionized water to wash off the residues of the remaining reagents as well as organic matter from the surface of the MPs. The rinsed items were then allowed to dry at room temperature. The procedure of qualitative analysis of MPs used in this study was also highly recommended by others [71,72,73]. However, according to available studies, microplastics identified in muscle tissues ranged from 1.2 to 0.45 µm [74]. Qualitative analysis of plastic items using an FT-IR ATR spectrometer at micrometric sizes is not possible [71,72]; therefore the identification of MPs inmuscle tissues was skipped in this study. Of the isolated plastic items, 146 were studied spectrally, which constituted 54% of all detected MPs.

Items were placed individually onto the ATR crystal. The FT-IR was run at a mid-range of 4000–400 cm^−1^ using 32 scans s^−1^ at 4 cm^−1^ resolution for each analysis. Air was used for the background spectrum. Spectrometer parameter settings used in this study were in agreement with the configurations used by others [75,76,77]. All spectra were compared and their identification was verified using an appropriate database (Hummel Polymer Sample Library) and software (Omnic Spectra software, ThermoFisher Scientific, Waltham, MA, USA). The spectra were interpreted based on spectral search, search by band positions, and visual match. In general, in accordance with EU recommendations [78], spectral matches of over 70% to the standard database were directly acceptable as MPs; however, in a very limited number of individual cases, polymers matchingthe reference spectra in the range between 70 and 60% were also accepted. A negligibly decreased limit of certainty was used only for samples characterized by features of synthetic polymers confirmed by detailed visual examination. Similar reasons for the slight lowering of the EU-recommended threshold were also used in other investigations [41,73,79,80].

### 2.5. Contamination Prevention

In each stage of fish rinsing, laboratory equipment washing, and reagent preparation, deionized water from a laboratory deionizer (HLP 10 UV, Hydrolab, Straszyn, Poland) was used. The deionized water source was originally equipped with a final filter with a mesh diameter of 0.2 µm (Sartorius stedim, Sartpore 2, Caps Size 4), which prevented water contamination with MPs items larger than 0.2 µm. The final filter was replaced before the study according to the manufacturer’s recommendations. The use of plastic materials was strictly limited in the research. Glass and steel laboratory equipment was used. Preparation equipment and glass vessels were triple rinsed with deionized water and covered with Al foil [81]. During the research, nitrile gloves and a cotton apron were worn to reduce the emission of fibers from the clothes [41,82]. Before starting the research, the laboratory tables were washed with deionized water and ethanol. As mentioned above, before preparation, each fish was carefully washed to remove any residual plastic items. Air contact with the sample during all laboratory procedures was minimized by covering the samples at each stage with a watch glass, aluminum foil, and a petri dish [83]. Simultaneously with a single test sample, a blind sample was prepared and treated according to an analogous protocol as the test sample. As a result, 87 blind samples were analyzed in this study.

### 2.6. Data Analysis

Due to the type of data collected, parametric analysis of variance was not possible. In particular, the idea of the use of two-way analysis of variance was abandoned since the data did not fulfil the parametric constraints. Alternatively, one criterion nonparametric analysis of variance method, namely the Kruskal–Wallis (K-W) test for multiple comparisons, was used [84] to compare the differences in both the absolute number and the relative percentage of MPs identified in samples according to categorical variables, such as species or organ type, separately. The K–W test was also used to assess the differences in the number of MPs using combinations of categorical variables (species vs.organs). However, in this mode, a test was performed independently for each species. Contingency tables [85], and the associated chi-square test and *p*-value, were used to check for significant differences in the relative frequency of the number of MPs both in separate organs according to the fish species (5 rows × 3 columns, df = 8) and in the overall tissues as far the colour of MPs according to morphological shapes (7 rows × 3 columns, df = 12). All tests were analyzed and considered significant at a *p*-value < 0.05. The statistical analyses were performed using TIBCO Statistica 13.3 software (TIBCO, Palo Alto, CA, USA).

## 3. Results and Discussion

### 3.1. General Microplastic Abundance

This study revealed that MPs were detected in all fish species. In 65 out of 87 specimens (74.7%), MPs were found in the gills, digestive tract, or liver. The number of MPs identified ranged from 1 to 12 items in individual fish, with an average of 4.09 items per fish. Identified items were representative of all particle sizes, not only of those of the largest cross-section higher than 500 µm. The cardinality of plastic items found in analyzed organs according to fish species is summarized in the contingency Table 2. Based on the results of the Kruskal–Wallis test for multiple comparisons, a difference in the number of MPs identified according to species was confirmed (H = 9.22, d.f.4). The lowest number of items was found in *Taurulus bubalis*, the moderate in *Gadus morhua*, whilethe highest was found in *Clupea harengus*, *Platichthys flesus*, and *Cyclopterus lumpus*; however, the observed ascending trend needs to be verified in future studies due to the low number of *Taurulus bubalis* caught as by-catch. Based on the counted values of the abundance of MPs, none of the statistical differences were revealed in the number of MPs found in various organs (H = 3.29, d.f.2) or using a combination of both categorical variables (species/organs). A lack of relative differences between pairwise grouped variables was also confirmed by the contingency table (Χ^2^ = 13.03, df = 8, *p* = 0.11).

However, it is worth emphasizing that apart from *Taurulus bubalis*, the number of items per fish in the case of benthic species was in the range between 4.6 and 5.6 for *Cyclopterus lumpus* and *Platichthys flesus*, respectively, while for pelagic species the range was between 2.8 and 3.7 for *Gadus morhua* and *Clupea harengus*, respectively.

None of the MP items were found in the blind samples. This suggests that the applied methods of contamination prevention were sufficient and none of the airborne contamination orwell as contamination from reagents took place. Moreover, the negative results of the analysis of the blind samples increased the confidence that the deionized water used in the sample and reagent preparation was not contaminated with MP items larger than 0.2 µm.

In comparison with recent data, studies on fish from the northern Baltic Sea indicate the lower content of MP items. An average abundance of MPs in all fish surveyed on the Swedish coast was 36.5% [41] and 50.4% in herring, in particular [42]. The percentage abundance of MPs found in herring and cod from Danish waters was 11% and 21%, respectively [43]. In the Bornholm basin, which is the northern basin of the Baltic Sea, MPs were identified in 20% of the examined herrings [37]. A lower concentration of MPs items in fish caught in the northern Baltic Sea seems to agree with expectations since most hazardous substances, including MPs, are released through large rivers (Neva, Odra, Wisła, and Niemen) into the waters of the southern and eastern Baltic. The mouths of these rivers are among the most polluted waters of the Baltic Sea and three of them are in its southern part.

Surprisingly, in the other study concerning the presence of MPs in fish caught in the southern Baltic Sea, the concentration of items was lower than in the current research. Białowąs et al. [44] identified items in herring and cod at 12.7% and 24.7%, respectively, while in this study it was 74% and 63%, respectively. It is suspected that such a large discrepancy may predominantly result from the procedure of fish acquisition, which in this case was commercial fishing using polymer-based fishing nets or, to a lesser extent, from the choice of different methods for determining MPs in fish. In the referenced study [44], synthetic fibers of the same chemical composition as airborne fibers were excluded from the analysis. In the current research, none of the MPs were found in blanks, eliminating their airborne origin. All items were found in fish, and even though the fibers can get into the water from the air they can still be swallowed by fish. In our opinion, the comparison of both sets of results obtained via the use of different methodological protocols could be ambiguous and this is why the current results are of great scientific soundness.

According to others, the abundance of MPs in fish caught in this research was higher than in fish caught in the Mediterranean Sea (2.36 items per fish) [86], in the southern Caspian Sea (2.29 items per fish) [87], in the North Sea (1.44 items per fish) [88], and in the Tokyo bay (3.06) items per fish) [89]. A similar abundance of MPs in fish was found in the Adriatic Sea (4.11 items per fish) [90], and in the East China Sea (about 4.0 items per fish) [91]. Our findings confirmed that fish samples captured from the southern BS were contaminated with MPs.

Based on the data presented above concerning the number of MPs identified in benthic and pelagic fish, *Platichthys flesus* was recognized as a massively MP-contaminated fish species. Microplastic items were detected in 89% of the tested flounders, with an average abundance of 5.6 items per fish. Our results show a higher contribution of MPs’ polluted flounders than in the studies presented by Bessa et al. [92], where plastic items were found in approximately 38% of the flounders’ specimens tested. However, having in mind some discrepancy of the quantitative data concerning *Platichthys flesus,* that might be related to the locations of the foraging area and the acquisition of specimens. In our opinion, the current results are in agreement with other recent studies reporting a higher abundance of MPs in benthic than pelagic fish species [74,93].

### 3.2. Accumulation of MPs in Tissues

The ability of MPs to penetrate from the digestive tract into edible tissues or organs of aquatic organisms has raised concerns about fish products; however, the majority of studies have discussed the abundance of MP loads in the digestive tract while few studies have investigated the presence of MPs in edible fish tissues, such as the liver.

**Figure 3:**
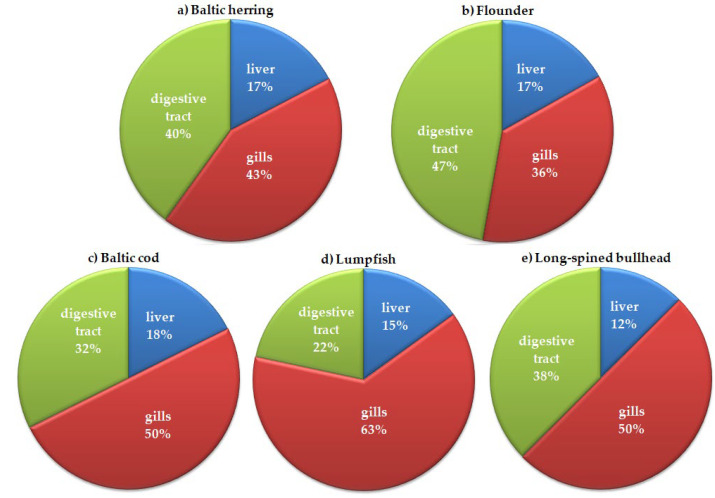
Percentage contribution of the numbers of MPs identified in the organs of five fish species caught for commercial purposes and as by-catch in 108 and 103 FAO areas in the southern Baltic Sea.

In the present research, MPs were observed in different fish organs, such as the liver, gills, and digestive tract of all five tested species.As mentioned above, the Kruskal–Wallis test for multiple comparisons performed on counted values did not confirm any statistical differences between MP abundance in organs; however, it did show a highly significant difference (H = 10.84; d.f.2, *p* = 0.004) between MP concentration in organs when percentage contributions were used instead of counted values. In general, the highest percentage contribution ranging between 36% and 63% was observed in gills, moderate in the digestive tract (22–40%), and lowest in the liver (12–18%) (Figure 3). The results presented in this study are in general in agreement with expectations when explaining the possible migration routes of MPs along the fish organs. Lu et al. [94] and Avio et al. [73] found some pieces of evidence confirming that MPs migrate from the digestive tract to the liver. However, they also concluded that plastic corpuscules of micrometric sizes can reach the liver both by penetration via the digestive tract and through the gills. We agree that the direct accumulation of plastic items (i.e., fibers, corpuscules, etc.) in fish gills due to respiratory activity or random processes, to some extent, complements the migration of MPs from the digestive tract to the liver.

**Figure 4:**
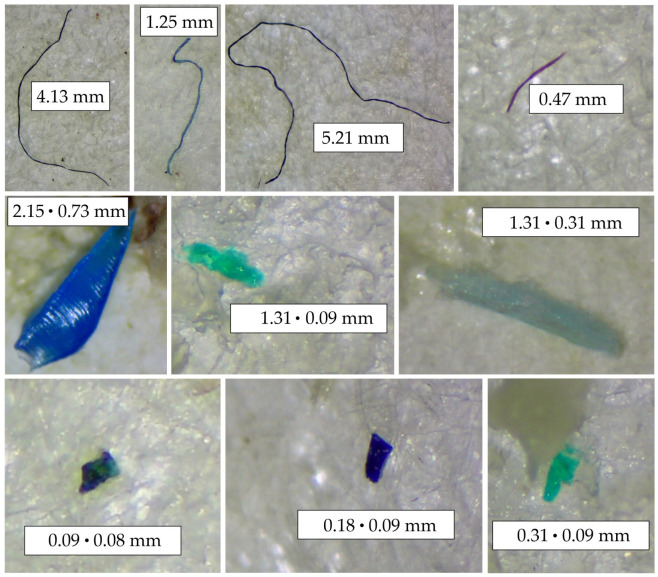
Images of MPs obtained by stereomicroscope coupled with a digital camera (description as 0.09 ⋅ 0.08 mm means max length × max width).

**Figure 5:**
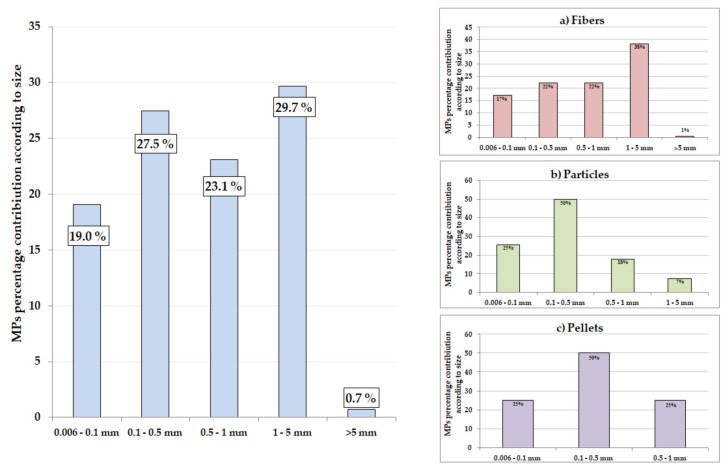
Categorized length dimensions for all morphological types of MPs identified in the Baltic Sea fish as well as corresponding frequency plots for fibers (**a**), particles (**b**), and pellets (**c**).

**Figure 6:**
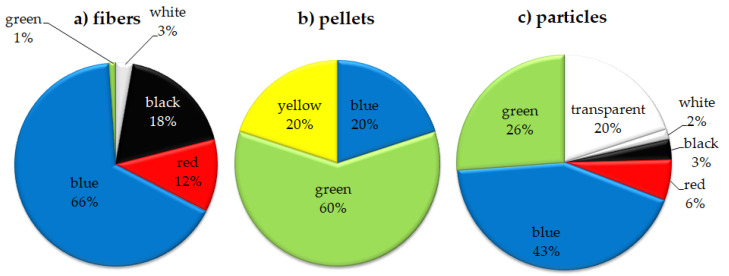
Percentage contribution of dominating colors of MPs for fibers (**a**), pellets (**b**), and particles (**c**)in the southern Baltic Sea fish.

The data displayed in Figure 3, Figure 4, Figure 5 and Figure 6 relate to all identified MPs (studied spectrally by the use of FT-IR and analyzed by optical microscope).

### 3.3. Physical Characterization

Typical examples of the microscopy images of MPs found in the organs of the BS fish are shown in Figure 4. According to the morphological shape, MPs were divided into three categories (particles, pellets, and fibers). Quantitative data concerning morphological shapes and colors of MPs found in fish organs are summarized in contingency Table 3.

Among the morphological types, fibers were prevalent among other forms (73.7%) followed by particles (24.4%), and pellets (1.9%). The contingency table separated yellow pellets from other morphological shapes and colors (Χ^2^ = 127.21, df = 12, *p* < 0.001).

The highest percentage contribution of fibers identified in organs is in agreement with other recent studies, where fibers were the dominant type of microplastics ingested by estuarine fish (>85%) [92,95,96,97,98,99] or by fish from other marine environments, such as deep-water habitats [100]. Ferreira et al. [101] and Scacco et al. [100] suggested that filaments may resemble natural food items for fish (nematodes, amphipods, and polychaetes) resulting in them mistaking them as prey. Jabeen et al. [102] concluded that freshwater systems are more likely to be contaminated by fibers because they could be released by wastewater treatment plants (WWTPs) [103,104]. For example, in the Solent estuary (UK) more than 80% of items collected in the water column were fibers [105]. Rivers can be an important source of fibers in marine ecosystems. Fibers can be also aggregated in the coastal and marine environment due to the fragmentation of fishing nets [98].Therefore, we conclude that apart from the fiber sources mentioned by others the highest percentage contribution of blue fibers might be caused by the procedure of fish acquisition that, in this case, was commercial fishing. Some plastic items released due to the use of fishing nets can be ingested by fish during trawling.

The average and median length and width of all identified pellets and particles were 0.39 mm, 0.28 mm, and 0.40 mm, 0.15 mm, respectively. The average length of fibers was 0.97 mm. MPs were identified in the size range from 0.06 mm to 5.21 mm (Figure 5). The identified plastic items were divided into five size ranges. The dominant size range was 1–5 mm (29.7%), then 0.1–0.5 mm (27.5%), and 0.5–1 mm (23.1%). Among the fibers, particles, and pellets, the dominant size range was 1–5 mm (38%), 0.1–0.5 (50%), and 0.1–0.5 (50%), respectively. A similar dominance of MPs with a length in the range of 0.5–5 mm was observed in wild fish from the North East Atlantic Ocean (where microplastics of size 0.5–1.5 mm accounted for >50%) [106]. The size contributes to the perception of prey by visual predators, and microplastics of a size comparable to prey are more likely to be actively swallowed by fish [106].

The most dominant color observed was blue (56.0%), then black (13.2%), transparent (10.5%), red (9.4%), green (8.3%), white (2.3%), and yellow (0.4%). The distribution of the colorof MPs according to morphological types is presented in Figure 6. Analogical colors of MP items were found in herring and cod caught in the southern Baltic [44]. The domination of blue items among the other colors is in agreement with findings presented by others [92,100,107,108,109]. Microplastic color is important since it may influence the visual uptake of food by fish. For example, fish from deep-water habitats often ingest mainly blue items, possibly because this resembles the color of the copepods they prey on [100,110]. Light and iridescent colors, which were also found here, could also resemble copepods and other prey that fish consume [111,112]. Lack of light transmission in benthic environments or even in shallow water may result in the accidental ingestion of transparent MPs.

### 3.4. Characterization of Polymers Using the FT-IR Technique

**Figure 7:**
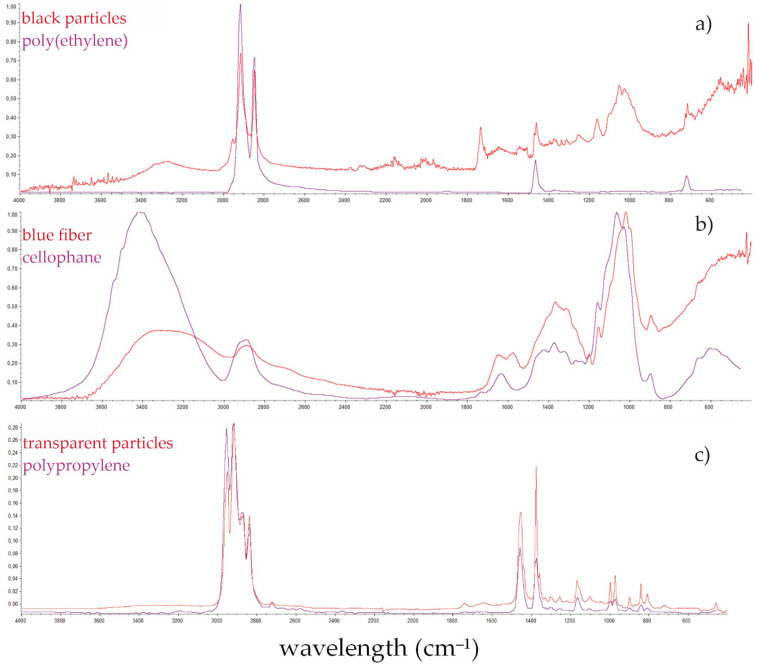
Sample spectra of identified polymers in fish: (**a**) polyethylene, (**b**) cellophane, (**c**) polypropylene.

The shapes of the recorded spectra did not perfectly fit the model spectra; however, they were generally in accordance with expectations. Figure 7 shows the spectra of the most commonly identified polymers.

As suggested by Song et al. [113] some additional interferences can arise from weathered plastics which may result in signals of slightly poorer quality than ideal ones from electronic databases. Moreover, particularly for cellophane, some previous research has shown that the degradation of cellophane in soil, e.g., with the participation of microorganisms, may affect the deformation of the FT-IR spectra peaks [114]. MPs isolated from the gastrointestinal tract, apart from being modified by environmental factors, are additionally degraded by the acidic pH of the stomach.

Qualification of polymer type allows some clues concerning the sources of the plastic debris to be obtained. A total of 54% of all identified MPs were subjected to chemical analysis by FT-IR, out of which 41% of MPs were cellophane (CP), 11% were identified as PE and polyamide (PA), while the contribution of polyester (PES) and polyethylene terephthalate (PETE) was 9% each (Figure 3). Similar results were obtained in the studies from China. CP (49.1%), PETE (10.6%), and PES (7.9%) were the common polymer types identified in the fish species from the Yangtze estuary, East China Sea, and South China Sea [102]. Moreover, in studies onthe Bohai Sea (China), CP was the most frequently identified polymer at a concentration of 56.8% [70]. Koongolla et al. [98] identified PE and PP in fish caught in Beibu Bay in the South China Sea at similar concentrations of 6%. A total of 93.5% of MP items subjected to FT-IR spectroscopic analysis were classified as polymers, as the percentage of the match to the reference spectra was above 70%. Only 10 (6.5%) items were in the 60–70% range, but they met the criteria of microscopic observation and were considered polymers.

The polymer composition diversity of MPs in fish might be derived from different sources of plastic pollution in marine environments. In the present study, CP was the dominant particle. Cellophane is used in industry as protective and decorative packaging, mainly for food. CP is a typical semi-synthetic material because a lot of additives are added to the products to increase their resistance to light and degradation. Due to many artificial ingredients, it shows low biodegradability though it is made of regenerated cellulose. CP as the biomass-derived polymer wasalso regarded as a microplastic in a recent report of UNEP [115] as well as in various previous studies [116,117]. PA (11%) was the next most abundant polymer found in tissues and organs of the BS fish. It is used as the timber of fishing nets used by artisanal fishermen [118]. PE was found in this study (11%), confirming that it is wide spread in the marine environment [41,119]. PE is widely used for manufacturing containers, dispensing bottles, wash bottles, and most commonly, plastic bags. PES was reported as a widely used polymer in textile and fishing gears [120]. Of all synthetic fabrics, PES stands out as the fibrous form of PETE, which is common in the marine environment and has been identified in this study. As confirmed by Pirc et al. [121], the domestic washing of textiles and garments is a constant and wide spread source of polyethylene terephthalate plastic microfibers into the environment. The authors estimated that during every wash, a long-fiber PES plush fleece releases 0.0012 wt% of loose microfibers into wastewater.

## 4. Conclusions

The results obtained in this study confirm that microplastics have infiltrated marine fish organs. MPs were dominantly identified in the gills and digestive tract. The general abundance of MPs in the studied organs of BS fish was similar to that in other studies. Among all morphological types, fibers were prevalent among other forms and were followed by particles and pellets. The dominant color of the MP was blue, but there was also red, black, transparent, yellow, green, and white. The majority of MPs found in Baltic fish were 1–5 mm. The FT-IR characterization revealed the presence of polymers predominantly containing CP, PE, PA, PES, PETE, and polyvinyl propionate. Fish guts and gills can be regarded as important organs in indicating the differences in MPs and monitoring ecological risks for fishes exposed to MP contamination. It is recommended to continue further research on the analysis of MPs in the tissues of marine fish, especially ediblefish.

## Figures and Tables

**Table 1:** Characteristics of different fish species caught as by-catch at the 108 and 103 FAO fishing zones.

Common Name	Species Name	Number of Caught Fish	Feeding Mode	Habitat	Mass (Mean ± S.D.) (g)	Mass (Range) (g)	Overall Length (Mean ± S.D.) (cm)	Overall Length (Range) (cm)
Baltic herring	*Clupea harengus*	27	plankton	pelagic	65.32 ± 18.39	34.43–108.04	22.8 ± 2.9	17.1–28.3
Baltic cod	*Gadus morhua*	19	fish	bentho-pelagic	297.66 ± 176.70	78.14–750.12	30.6 ± 4.8	20.8–41.8
Flounder	*Platichthys flesus*	18	plankton, insect larvae, adult annelids, mollusks, crustaceans, and small fish	benthos	171.91 ± 69.30	34.42–264.05	25.0 ± 4.4	17.1–33.6
Long-spined bullhead	*Taurulus bubalis*	6	polychaetes, decapods, mollusks, and small fish	benthos	284.61 ± 208.58	80.73–650.35	23.3 ± 3.1	17.8–26.8
Lumpfish	*Cyclopterus lumpus*	17	crustaceans, jellyfish, and small fish	benthos	165.54 ± 39.04	110.06–271.70	15.5 ± 1.3	13.9–18.8

Note: S.D.—standard deviation.

**Table 2:** Percentage contribution of fish with MPs identified and the distribution of plastic items according to the type of fish organ.

Species Name	N_all_ N_MPs_	Contribution of Fish with MPs Found (%)	MPs (Items)			Total
			Liver	Gills	Digestive Tract	
*Clupea harengus*	27 20	74%	13	32	30	75
*Platichthys flesus*	18 16	89%	15	32	42	89
*Gadus morhua*	19 12	63%	6	17	11	34
*Cyclopterus lumpus*	17 13	76%	9	38	13	60
*Taurulus bubalis*	6 4	67%	1	4	3	8
Total			44	123	99	266

Note: N_all_—total number of specimens, N_MPs_—number of specimens with MPs identified in their organs.

**Table 3:** Color and shape of MPs found in various organs of the Baltic Sea fish.

Color	Particles	Pellets	Fibers	Total
transparent	13	0	15	28
white	1	0	5	6
black	2	0	33	35
red	4	0	21	25
blue	28	1	120	149
green	17	3	2	22
yellow	0	1	0	1
Total	65	5	196	266

## Data Availability

The data presented in this study are available on request from the corresponding author. The data are not publicly available due to the fact they will be a part of Ph.D. thesis.
